# Development of an Immunodiagnostic Test for Screening Human Brucellosis Cases Using the Whole-Cell Antigens of Brucella abortus

**DOI:** 10.21315/mjms2020.27.6.3

**Published:** 2020-12-29

**Authors:** Nidhi M Bhartiya, Aliabbas A Husain, Hatim F Daginawala, Lokendra Singh, Rajpal S Kashyap

**Affiliations:** Biochemistry Research Laboratory, Central India Institute of Medical Sciences, Nagpur, India

**Keywords:** brucellosis, indirect ELISA, whole-cell antigens, diagnosis

## Abstract

**Background:**

Human brucellosis is an important zoonotic disease of public health and often remains neglected owing to lack of sensitive and efficient diagnostic methods. This study evaluates diagnostic utility of in-house designed enzyme-linked immunosorbent assay (ELISA) using whole-cell antigens of *Brucella abortus* (*B. abortus*) S19 against the commercially available kits.

**Methods:**

A prospective cohort study involving different populations within the Vidarbha regions of Maharashtra, India was conducted through camps organised from May 2009 to October 2015. A total of 568 serum samples were collected from high-risk people recruited as study cohorts based on inclusion criteria, additional risk factors and clinical symptoms. Samples were evaluated by indirect ELISA using the whole-cell antigens of *B. abortus*. The results were compared with the commercially available IgG detection ELISA kit to ascertain the specificity and sensitivity of the developed test.

**Results:**

Fever, body ache, joint pain, lower back pain, loss of appetite and weight loss were major symptoms associated with the disease. With the cut-off of > 0.8, the positivity of brucellosis infection was at 12.32% (70/568) compared to 9.33% (53/568) as detected by the commercial kit. The in-house developed ELISA method yielded a sensitivity of 87.5% and specificity of 99.18% as compared to the commercial kits (sensitivity −80.30% and specificity −99.6%).

**Discussion:**

The *B. abortus* S19-derived whole-cell protein-based ELISA is rapid and cost-effective and can be used for screening brucellosis infection in lieu of the commercially available ELISA kits.

## Introduction

Human brucellosis is an important zoonotic disease reported worldwide. Human transmission of the *Brucella* infection is through direct exposure to an infected domestic animal or by the consumption of raw milk and milk products (1). It is prevalent in rural communities where a majority of people lives in proximity to the animals (2). An occupational association to disease transmission in meat-eaters, zookeepers, animal handlers, farmers and veterinarians has also been reported. Human brucellosis can cause a range of symptoms similar to flu or other viral infections, often remaining neglected due to a lack of prompt diagnosis.

Despite the availability of effective therapeutic interventions, the recent past has seen an uncontrolled rise in human brucellosis, particularly in areas of high endemicity. Various reports of brucellosis as a commonly encountered disease in veterinarians are available, citing a prevalence rate of 17%–34% (3, 4). Furthermore, these numbers represent compromised figures due to a lack of effective diagnostic tools and limited epidemiological information.

Existing tests for diagnosis of brucellosis comprise of microbiological, molecular and serological tests like the Rose Bengal test (RBT) and serum agglutination tests (SAT) (5, 6). Despite being cost-effective and offering rapid diagnosis as compared to the standard culture and molecular methods, these tests lack the necessary sensitivity and specificity and often fail to discriminate between the true-positive and false-positive serological results (7). Blood culture has been a recognised gold standard for the confirmation of brucellosis. However, culturing methods are laborious, less sensitive and require elaborate biosafety facilities that are beyond the scope of most diagnostic facilities in brucellosis endemic regions (8). The latest researches show that enzyme-linked immunosorbent assay (ELISA) is more reliable for diagnosing *Brucella* infection when compared to RBT and SAT (6). ELISA is capable of readily identifying the individual IgM and IgG antibody to the surface antigen of *Brucella abortus* (*B. abortus*), permitting a better clinical correlation (6).

Many diagnostic tests using various *Brucella* antigens have already been developed. Unfortunately, the test results are variable in terms of sensitivity and specificity, limiting their utility in serodiagnosis (9, 10). Earlier studies by Al Dahouk S and colleagues (11) discovered the immunoproteomic characterisation of *B. abortus* 1119-3 preparations used for the serodiagnosis of *Brucella* infections. Hitherto, no study has analysed the diagnostic capacity of whole-cell antigens of the *Brucella* S-19 strain for diagnosing human brucellosis infection. The whole-cell proteins of a bacterium contain a cocktail of different immunodominant antigens with regulatory and functional roles. Additionally, they are more immunogenic for inducing both IgG and IgM responses compared to individual antigens, thereby justifying their diagnostic utility in serodiagnosis (12).

The present work evaluates the diagnostic utility of in-house designed ELISA using whole-cell proteins of *B. abortus* S19 as compared to commercially available tests. The main purpose was to develop an improved and inexpensive test for the diagnosis of brucellosis infection in humans.

## Methods

### Study Population and Study Design

A prospective cohort study was carried out in a zone of high brucellosis endemicity within the Vidarbha region of Maharashtra. A total of 1142 participants were recruited through camps organised from May 2009 to October 2015 based on predefined inclusion criteria, with added risk factors like unconventional food intake habits (consuming raw milk/food products) and animal exposure. The associated clinical symptoms included fever, joint pain, joint swelling, chest pain, headache, back pain and night sweating. All the participants were verbally explained about the study before enrolling and subsequent written consent was taken from all the recruited participants. A detailed medical history of the recruited individuals was collected using a structured questionnaire prepared beforehand by a team of expert clinicians and scientists. Baseline factors like age, gender and other risk factors were recorded. Samples were collected from different population and study zones (suspected of *Brucella* endemicity) categorised as follows:

### Group 1: Malnourished Population with High Exposure to Animals

The recruited participants from this group were mostly tribal from different villages of Melghat, Vidarbha living closely with their livestock. The tribal populations had poor socioeconomic and living conditions and relied on farming and animal rearing as the major occupation.

### Group 2: Meat Sellers from the High Endemic Area

This recruited group were from a specific area of the Nagpur district in the Vidarbha region of Maharashtra. Most of the recruited participants were meat shop owners or labourers working in a meat shop involved in slaughtering (cows and goats) routinely.

### Group 3: Zookeepers and Animal Handlers

This group included zookeepers, veterinarians and dairy farmers from a specific locality within the Nagpur district. The participants were involved in guarding, cleaning, feeding, and taking care of animals in the zoological garden. Veterinarians deal with health checkups, treatment and post-mortem of diseased animals. The associated risk factors for brucellosis were expected to be higher in this group.

### Group 4: Farmers

Farmers from different villages of the Vidarbha region who had agriculture as their major occupation along with animal rearing (cattle and goats) belonged to this group. The farmer or members of the family were personally involved in the care of these animals along with milking and delivery of pregnant animals. These activities were assumed as possible risk factors for contracting brucellosis in these individuals.

**Figure 1:**
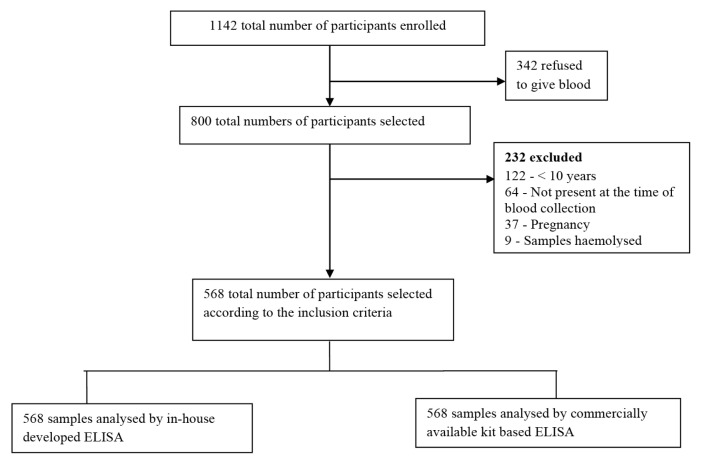
Study flow diagram for participant recruitment

Out of the 1142 participants, 342 refused to give blood. Of the remaining 800 participants, 232 were further excluded according to the exclusion criteria — pregnant women (*n* = 37), children below 10 years of age (*n* = 122) and participants who were not present at the time of blood collection (*n* = 64). Few samples that got hemolysed (*n* = 9) were also not included in the study. A total of 568 participants matching inclusion criteria were finally recruited for the study (Figure 1).

### Collection of Blood Samples

Approximately 2 mL of blood was collected in a plain vacutainer tube and allowed to clot at 37 °C to obtain the serum. A total of 500 μL serum was separated and stored at 4 °C until further analysis. A separate 5 mL of venous blood was collected for culture and added aseptically into the *Brucella* broth (HiMedia, Mumbai, India). The medium was incubated at 37 °C under 5% CO2 for 30 days and examined for growth. The collected samples from each patient were parallelly processed for serology and culturing. Identification of *Brucella* strains was carried out using standard classification tests comprising of growth characteristics, Gram staining, a modified Ziehl–Neelsen stain, oxidase activity and urease activity. A case of brucellosis was defined having either blood culture and/or ELISA positive individually or in combination. This criterion was used for calculating the sensitivity and specificity of the ELISA.

### Preparation of B. abortus Culture Whole-Cell Antigens

The pure culture of *B. abortus* S19 was a kind gift from Dr Nitin Kurkure (Nagpur Veterinary College, Maharashtra). The bacteria was further subcultured in 1 L of *Brucella* broth (BD Life Sciences, USA) and kept at 37 °C for 7 days with a 5% CO_2_ requirement.

The protocol by Corrente et al. (13) with some modifications was followed for the isolation of whole-cell antigens. Briefly mid-log phase culture of *Brucella* S19 strain (O.D ~ 0.9–1.0) was centrifuged at 10,000 rpm for 10 min. The pellet was re-suspended in ice-cold 1× sterile phosphate-buffered saline (PBS) and centrifuged again at 10,000 rpm for 10 min. This step was repeated thrice to ensure thorough washing of the cells traces of media. The pellet was then suspended in the cell-lysis buffer (bacterial protein-extraction buffer, Thermo Fisher Scientific, USA) and incubated at room temperature for 20 min, followed by sonication for 2 min with a 15-sec pulse (each) for on and off. Post-sonication, supernatant was recovered by centrifugation at 7280 g for 10 min. The supernatant was dialysed in a 1× sodium-phosphate buffer and harvested. The sample was subjected to protein quantification using the Qubit fluorometer. The resultant whole-cell antigens were lyophilised and refrigerated until further use.

### Serological Tests

Indirect ELISA was performed according to Kashyap et al. (14) described elsewhere. Ninety-six-well polystyrene microtitre plates were coated with different dilutions of bacterial-protein extract (20 ng/100 μL) and incubated at 37 °C for 3 h. The wells were then washed once with PBS in Tween 20 (PBST) and blocked with 100 μL of blocking buffer (0.5% bovine serum albumin in PBS) and incubated at 37 °C for 2 h. After blocking the wells were washed thrice, followed by the addition of the serum sample (1:400 dilutions in 1× PBS). After 35 min of incubation at 37 °C, the wells were washed thrice with PBST followed by the addition of a secondary antibody — goat antihuman IgG conjugate (1:20,000 dilution in PBS) and incubated for 30 min at 37 °C. Further, after this incubation, the wells were again washed four times with PBST with the subsequent addition of 100 μL of enzyme-substrate tetramethylbenzidine/hydrogen peroxide (TMB/H_2_O_2_) and incubated at room temperature for 3 min. The reaction was stopped by adding 100 μL of 2.5 N H_2_SO_4_, and absorbance of colour in each well was read at 450 nm.

### ELISA by the Novatec Kit

Detection of IgG antibody by ELISA was performed using a commercial kit [Novatec Immunodiagnostic GmbH, Germany] as per the manufacturer’s instructions. A 100 μL of diluted serum sample (1:100) and ready-to-use positive control, negative control, cut-off and diluent blank were added to the microtitre wells coated with the antigen. The samples were then incubated at 37 °C for 60 min, with subsequent washing of the wells three times. Later, an anti-human IgG antibody conjugated with an enzyme (horseradish peroxidase) was added and incubated for 30 min at room temperature. All wells were then washed to remove excess conjugate and 100 μL of enzyme-substrate (tetramethylbenzidine/hydrogen peroxide) was added afterward and incubated for exact 15 min at room temperature in the dark. Finally, the reaction was stopped by adding 100 μL of 2.5 N H_2_SO_4_. The enzyme reaction with the substrate gives a coloured product. The colour intensity is proportional to the IgG and can be quantified using the photometric methods. IgG titres above 0.6 OD units (as per kit cut-off values) were considered positive. Sensitivity and specificity, as given by the manufacturer were more than 95%.

### Calculation of Sample Size

The prevalence of brucellosis has been reported to be 15% (4). Referring to this prevalence and considering a tolerable margin error as 3%, a sample size of 545 would provide the true estimate of prevalence with 95% confidence and 80% power of test.

### Statistical Analysis

The frequencies (%) of the demographic, behavioural and clinical factors were measured on a nominal scale. Statistical analysis was performed using the MedCalc statistical software (version 10.1.2.0) and *P* < 0.05 was considered statistically significant. Test concordance was assessed using the Kappa (k) statistic. Positive predictive values (PPV) and negative predictive values (NPV) were calculated using a diagnostic test evaluation (2 × 2 table) (MedCalc 10.1.2.0).

## Results

Out of the 1142 eligible participants, data from 568 participants were eventually considered for the final analyses. The baseline characteristics of the study population are described in Table 1. The majority of the recruited participants belonged to the age group of 18–38 years (median age of 35.6 years) with a slightly higher male (58%) ratio than females (42%). Among the recruited populations, a large proportion was exposed at risk owing to the consumption of raw milk and exposure to animals. Symptoms like body ache, joint pain, lower back pain, loss of appetite and weight loss were significantly present in all recruited cases.

A checkerboard titration method was used to optimise ELISA, wherein different concentrations of antigens can be tested against different sample concentrations at once. The final selected concentration had the least reactivity of infectious controls (tuberculosis positive, dengue positive, Chikungunya positive and *Yersinia* spp. positive) compared to the culture-positive *Brucella* serum sample. The optimum concentration of the sample, secondary antibody and whole-cell antigen was selected as 1 μL of serum sample in 400 μL of 1× sterile PBS, 1:20,000 and 20 ng/100 μL of 1× sterile PBS, respectively (Table 2). The healthy controls were devoid of any sort of infection. Table 3 shows the data for a positive brucellosis infection in the study population as detected by the in-house developed ELISA protocol and commercial Novatec kit. With a cut-off value > 0.8, the positivity of brucellosis infection as detected by the new kit was at 12.32% (70/568) compared to 9.33 % (53/568) as detected by the commercial kit (cut-off value > 0.6). Cut-off for the same was calculated based on the titres obtained in 10 culture-positive brucellosis samples.

**Figure 2:**
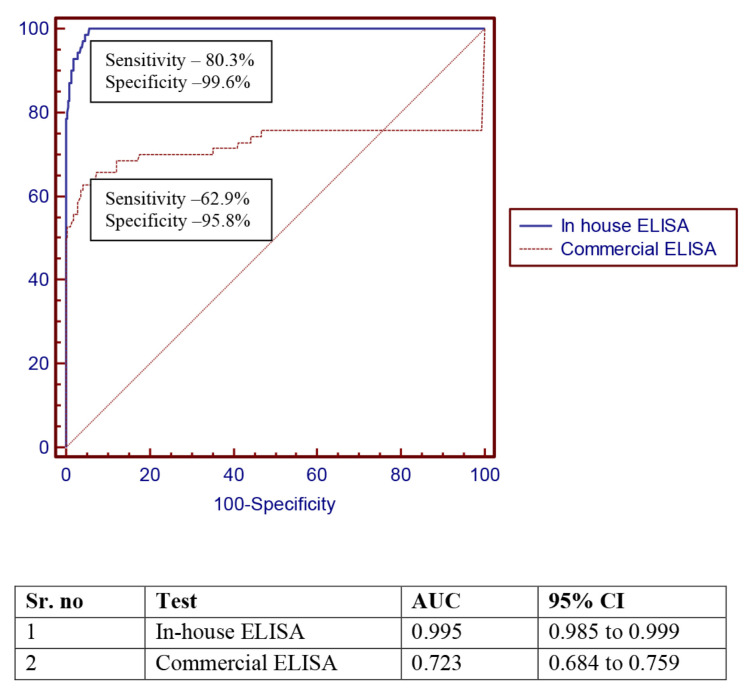
Comparative ROC analysis for all samples (overall) in the study population. The ROC plots the true-positives rate (sensitivity) against the false-positives (100-specificity)

Figure 2 shows the Receiver Operating Curve (ROC) analysis for both the evaluated tests in the study population. With a cut-off value of > 0.8, the in-house developed ELISA method yielded a sensitivity of 87.5% and specificity of 99.18% as compared to the commercial kit, which showed a sensitivity and specificity of 80.30% and 99.6%, respectively. Although the in-house test yielded a slighter better sensitivity, the positive and negative predictive values were essentially similar for both kits (Table 4).

Table 5 shows concordance and positivity results for the diagnosis of brucellosis by both in-house ELISA and commercially available kits. Higher concordance (> 90%) between the two tests was found for diagnosing brucellosis in the study population. The concordance between both tests in different study groups was found to be 96.29% in meat eaters, 95.90% in malnourished group, 97.80% in zookeepers and 96.25% in farmers, respectively. On comparing the culture results with ELISA, it was found that the culture positivity was quite low, indicating a low sensitivity of the culture method for *Brucella* diagnosis.

## Discussion

Brucellosis, a major zoonotic infection in humans, especially in developing countries (15), is often misdiagnosed or under-diagnosed due to overlapping clinical manifestations with many bacterial infections, therefore the need for presumptive screening to support the diagnosis and initiate therapeutic interventions. The laboratory confirmation of human brucellosis is based on microbiological, serological or molecular methods, each having its advantage and disadvantage (16, 17, 18). The commercially available serological kits are based on IgG-detection in sera of brucellosis cases. Although these kits produce rapid results, their use in low-resource settings is limited by high costs. To overcome the existing diagnostic constraints and develop a rapid, cost-effective test for the detection of human brucellosis, we have developed an in-house ELISA test using the whole-cell lysate of a smooth strain of *B. abortus*, the S19 strain. The diagnostic utility of the developed ELISA kit was evaluated in the high-risk occupationally-susceptible populations and compared the results with the commercially available diagnostic kit. Based on the comparative diagnostic utility, the in-house developed ELISA method yielded better sensitivity and specificity compared to the commercial kit.

The detection of antibodies against the lipopolysaccharide portion of the Brucella spp. has been the pillar of most serodiagnostic methods for brucellosis screening. However, such tests are at a risk for false-positive reactions with other related pathogens, especially Yersinia enterocolitica O:9, which has shown to have a high cross-reactivity with the *Brucella* spp. (19, 20). However, in certain studies, the researchers have tried using the recombinant cocktail proteins of the outer membrane of Brucella spp. for the serodiagnosis of brucellosis. Interestingly, such methods have yielded promising results with good sensitivity and specificity (21, 22, 23). The sensitivity and specificity of the in-house developed kit could not be compared with the aforementioned kits since the developed kit uses whole-cell antigens of B. abortus as opposed to other kits that are based on a single antigen.

Immunoproteomic analyses have identified an array of numerous immunodominant proteins present exclusively in the whole-cell lysate of *B. abortus* having regulatory and functional roles, and are more immunogenic for inducing both IgG and IgM responses (24, 25). The use of the indigenous ELISA, based on whole-cell antigens, has already provided commendable results in diagnosing bovine brucellosis (13). Moreover, when compared, the developed ELISA demonstrated better sensitivity and specificity over conventional tests like RBT, thereby reducing the demand for additional serological tests. This simple modification of using combined antigens of the whole-cell, instead of individual antigens, makes this technique close to an ideal test for the serodiagnosis of brucellosis, which can potentially be used for quick screening of suspected cases in small-scale laboratories. Consequently, a combined approach of using whole-cell antigens was used for the diagnosis of human brucellosis. In this study, a high concordance between both commercial and the in-house developed ELISA kits was found, thereby indicating that the newly developed assay can be used to replace the commercial kits, for reducing the cost of diagnosis. *Brucella* spp. could be isolated from 28 samples only, substantiating that the sensitivity of *Brucella* blood-culture is low as compared to the in-house developed test and the commercially available test which could detect more positive cases. Interestingly, the culture proved negative despite a high titer of IgG antibodies in serum samples. However, ELISA also recorded high IgG antibodies, indicating good diagnostic utility since IgG could be due to past infection.

Our results illustrated that the majorly affected groups were the farmers, veterinarians, animal handlers, slaughterhouse workers and meat eaters, which is in agreement by earlier, studies by Pathak and colleagues (26). However, it is also important to emphasise that the diagnosis of human brucellosis has to be made on a combination of compatible symptoms, risk factors, clinical findings, and detailed patient investigation. A detailed survey revealed that the major symptoms associated with the disease were fever, body ache, joint pain, lower back pain, loss of appetite and weight loss with a significant association with risk factors like consumption of raw milk and exposure to animals, where these findings correlated well the other authors (27–31).

While these results are preliminary, the test developed has a high potential for serodiagnosis of brucellosis. Likewise, the B-cell epitopic regions or the antigenic determinants of the immunodominant proteins of the cell lysate could also be developed to be further evaluated for the development of a more specific and sensitive test. The present study needs further evaluation in larger cohorts for validation and implementation.

## Conclusion

A novel, simple, rapid and cheaper ELISA method based on the whole-cell proteins of the *B. abortus* S19 as antigens was developed. This assay could be used for screening of brucellosis infection and can be used in lieu of the commercially available ELISA kits, culturing and molecular tools that are time-consuming and costly. Rapid and sensitive screening of high-risk populations, who are occupationally susceptible, could help contain the spread of the diseases and implement treatment strategies early.

## Figures and Tables

**Table 1:** Baseline characteristics of the study population

Sr. No	Baseline characteristics (n = 568)	Malnourished (*n* = 297)	Meat eaters (*n* = 146)	Zookeepers and animal handlers (*n* = 45)	Farmers (*n* = 80)	Chi-square test	P-value
1	Age groups						
	< 18	34 (11.4)	20 (13.7)	0	0	3.13	0.078
	18–28	79 (26.6)	32 (21.9)	2 (4.4)	18 (22.5)	100.847	< 0.001
	28–38	92 (31)	28 (19.1)	8 (17.8)	20 (25)	114.486	< 0.001
	38–48	19 (6.4)	29 (19.9)	7 (15.6)	13 (16.3)	15.529	0.014
	48–58	41 (13.8)	14 (9.6)	24 (53.3)	21 (26.2)	15.76	0.013
	> 60	32 (10.8)	23 (15.7)	4 (8.9)	8 (10)	30.493	< 0.001
2	Gender						
	Males	172 (57.9)	64 (44)	42 (93.3)	49 (61.3)	135.936	< 0.001
	Females	125 (42.1)	82 (56)	3 (6.7)	31 (38.7)	146.037	< 0.001
3	Signs and symptoms						
	Fever	76 (25.6)	21 (14.4)	11 (24.4)	29 (36.25)	72.606	< 0.001
	Chills	5 (14.85)	3 (2.05)	0	4 (5)	0.5	0.778
	Abdominal pain	14 (41.6)	18 (12.3)	0	1 (1.25)	14.364	0.008
	Chest pain	12 (35.6)	32 (21.9)	0	17 (21.25)	10.656	0.049
	Body ache	61 (20.5)	24 (16.4)	7 (15.5)	30 (37.5)	50	< 0.001
	Headache	32 (10.8)	21 (14.4)	8 (17.8)	13 (16.25)	17.784	0.005
	Joint pain	49 (16.5)	15 (10.3)	5 (11.1)	30 (37.5)	44.475	< 0.001
	Lower back pain	36 (12.1)	21 (14.4)	2 (4.4)	12 (15)	35.197	< 0.001
	Night sweating	4 (1.3)	2 (1.4)	3 (6.7)	3 (3.75)	0.667	0.881
	Nausea	2 (0.67)	0	1 (2.2)	1 (1.25)	0.5	0.788
	Vomiting	2 (0.67)	4 (2.7)	3 (6.7)	7 (8.75)	3.5	0.320
	Loss of appetite	65 (21.9)	4 (2.7)	1 (2.2)	3 (3.75)	159.932	< 0.001
	Weight loss	72 (24.2)	3 (2.05)	2 (4.4)	11 (13.75)	153.727	< 0.001
4	Risk factors						
	Consumption of raw milk	92 (31)	34 (23.4)	3 (6.7)	9 (11.25)	143.449	< 0.001
	Exposure to animals	152 (51.2)	89 (61)	35 (77.8)	48 (60.0)	102.593	< 0.001

**Table 2:** Standardisation of the protocol for antibody detection in serum samples using the whole-cell proteins of *B. abortus*

Samples	Antibody concentration	Antigen concentration (ng/uL)			
		5 ng/100 uL	10 ng/100 uL	15 ng/100 uL	20 ng/100 uL
*Brucella* culture positive sample	1:5,000	0.76	1.18	1.28	1.62
	1:10,000	0.84	0.82	0.87	1.6
	1:20,000	0.72	1.51	1.11	1.63
Healthy control	1:5,000	1.47	0.34	1.34	1.26
	1:10,000	0.72	0.75	0.75	0.56
	1:20,000	0.64	0.65	0.65	0.49
Tuberculosis positive	1:5,000	1.23	0.24	1.32	0.59
	1:10,000	0.55	0.66	0.36	0.4
	1:20,000	0.23	0.21	0.32	0.41
Dengue positive	1:5,000	0.23	0.33	0.49	0.41
	1:10,000	0.52	0.32	0.32	0.32
	1:20,000	0.26	0.12	0.23	0.26
Chikungunya positive	1:5,000	1.0	1.02	1.11	0.89
	1:10,000	0.52	0.65	0.82	0.60
	1:20,000	0.35	0.24	0.36	0.42
*Yersinia* spp. positive	1:5,000	0.92	0.84	0.96	0.65
	1:10,000	0.52	0.36	0.33	0.33
	1:20,000	0.4	0.23	0.41	0.33

Notes: The optimum concentration of antigen was taken to be 20 ng while that of the antibody was 1:20,000 where infectious control 1, infectious control 2, infectious control 3 and infectious control 4 being tuberculosis positive, dengue positive, Chikungunya positive and *Yersinia* spp. positive, respectively. Healthy controls were devoid of any sort of infection

**Table 3:** Comparison of the cut-off values of two tests between in-house ELISA tests using whole cell antigens of *B. abortus* S19 and commercial Novatec IgG ELISA kit

Sr. no	Test	Total (*n*)	Cut-off	OD Range (450 nm)	Positive (*n*)
1	In-house ELISA	568	> 0.8 OD units	0.8 OD units – 1.523 OD units	70
2	Novatec ELISA	568	> 0.6 OD units	0.6 OD units – 2.032 OD units	53

Notes: OD = optical density

**Table 4:** The sensitivity, specificity, PPV and NPV between in-house developed ELISA assay and commercial Novatec IgG ELISA kit

Sr. no	Test	Sensitivity (95% CI)	Specificity (95% CI)	PPV* (95% CI)	NPV** (95% CI)
1	In-house ELISA	87.5%	99.18%	94.59%	97.98%
2	Novatec ELISA	80.30%	99.6%	96.36%	97.47%

Notes:

* Positive predictive value (range - 87.45%–99.45%);

** Negative predictive value (range - 95.70%–98.64%);

CI – Confidence interval

**Table 5:** Overall positivity of IgG detection of in-house and commercial kit based ELISA results for all the study population

Sr. no	Population	Number (*n*) Total 568	In-house ELISA positive (%)	Novatec IgG ELISA positive (%)	Culture positive (%)	Concordance of in-house ELISA and Novatec ELISA (%)
1	Malnourished	297	22 (7.4)	13 (4.37)	05 (1.7)	96.29%
2	Meat eaters	146	21 (14.3)	15 (10.27)	09 (6.1)	95.90%
3	Zookeepers and animal handlers	45	2 (4.44)	3 (6.66)	06 (13.3)	97.80%
4	Farmers	80	25 (31.25)	22 (27.5)	08 (10)	96.25%
